# Carbolic Acid in Scarlatina

**Published:** 1869-04

**Authors:** H. T. Cleaver

**Affiliations:** Professor of Obstetrics and Diseases of Women and Children, College of Physicians and Surgeons, Keokuk, Iowa


### CARBOLIC ACID IN SCARLATINA.

BY H. T. CLEAVER, M. D.,

Professor of Obstetrics and Diseases of Women and Children, College of Physicians and Surgeons, Keokuk, Iowa.

During the months of November and December, 1868, scarlet fever prevailed to a greater extent than in any one of the preceding twenty years of my practice in Iowa; and although mild in its general type, in many cases the throat and symptoms were such as usually present in the severer forms of that disease.

The success attending my treatment of over seventy patients, with but one fatal case, (and that one an infant of strumous habits,) inspire me with a great deal of confidence in the efficacy of carbolic acid as a means of greatly lessening the mortality of this dreadful disease.

Without any disposition to ascribe undue power to this (to me) new therapeutic agent in this disease, I respectfully submit the manner of its use, with the following results :

I have given it in all stages of the disease, and in many cases relied upon it as the sole therapeutic means employed, and realized the same favorable results as where other supposed valuable remedies were associated with it in the treatment.

The following is my usual prescription :

*R.* Acid Carbolic, 1 drachm; Dilute Alcohol, 2 oz.

Mix a teaspoonful of this to a tablespoonful of water, used either as a gargle or with the mop, depending upon age and ability to gargle—say once every two hours. Of the same give 10 to 20 drops in Mur. Acacia at same intervals; dose depending upon age of patient.

I have been as much surprised as gratified at the almost immediate relief afforded by the application of this to the inflamed throat. I have repeatedly tested the comparative value of this and the Chlorate Potass and Chlor. Iron, with unvarying results in favor of the Acid as a palliative and curative application. The only unpleasant symptom I have observed after its use in greater strength, is a temporary impairment of power of deglutition. Whether this condition was due entirely to the acid, or depended somewhat upon other causes, is a subject for more extended observation. I have not observed this symptom to supervene upon the use of any other topical remedy.

That it has the power of lessening the exalted action of the hear and quieting the delirium, so uniformly met with in Scarlatina, I have no doubt. That it modifies the action of the scarlatina poison, and to some extent averts the disease, I am much inclined to believe. I have also given it as a prophylactic in families where the disease has made its appearance with the apparent effect of ameliorating the severity of the attack.

In none of the cases where it has been used have dropsical effusions occured as a sequel, such as I have seen in cases treated with other remedies.

